# Putting women's eyesight first

**Published:** 2016

**Authors:** Damian Facciolo, Camille Neyhouser

**Affiliations:** Regional Programme Manager (Western Pacific): International Agency for the Prevention of Blindness, Singapore.; Learning and Best Practice Coordinator & Gender Strategy Coordinator: The Fred Hollows Foundation, Sydney, Australia.

**Figure:**
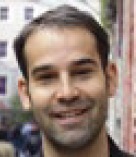
Damian Facciolo

**Figure:**
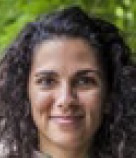
Camille Neyhouser

Two-thirds of blindness and visual impairment occurs in women^1^, and recent prevalence surveys in Vietnam and China show an imbalance in the coverage of services across some areas. We know that, compared to men, women account for the greater burden of blindness from cataract.^2^ Women and girls face numerous barriers in accessing eye care services and are less likely to utilise them.

Understanding gender dimensions is an important aspect of public health and development. In 2009, Gender was the theme for World Sight Day and the focus of articles in this Journal.^3^ The World Health Organization action plan called Universal Eye Health: A Global Action Plan 2014–2019' prioritises equity and it is clear that we need to do more to improve services for women and girls.

## What are the barriers for women and girls?

Often, the barriers faced by women are not fully understood by health care providers. Greater understanding can be achieved through regular data collection (separately for men and women), knowledge, attitudes and practice (KAP) surveys, satisfaction surveys and gender analyses. Consultation with both women and men helps to ensure that services are delivered in a way that is gender-sensitive and relevant to that community.

Lack of access to household resources and opportunity costs (e.g. loss of income due to the time taken to attend appointments) prevent women from accessing eye care services. As women are often less educated than men and are less likely to access information outside the home due to their caretaking role, they are also less aware of eye health treatments and services. Women have reduced power to make decisions in the household and sometimes de-prioritise their own health in favour of others in the family.

**Figure:**
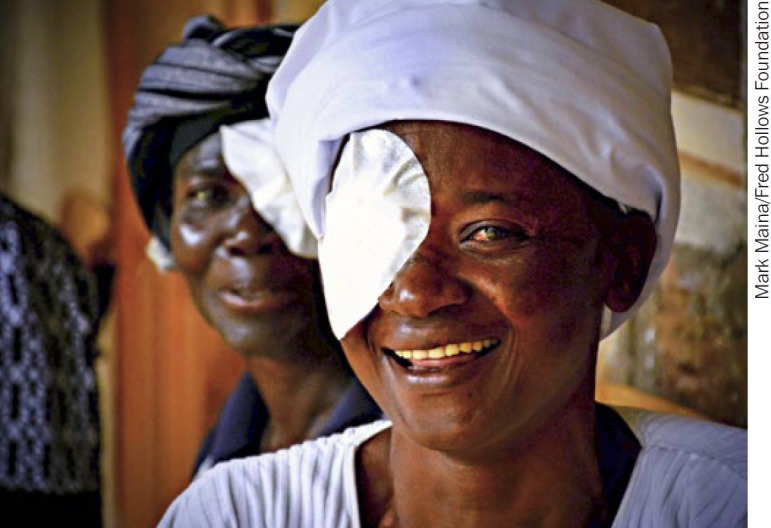
A woman waits to have her patch removed after surgery. KENYA

## How can we improve?

Although many barriers exist for both men and women, gender inequalities makes access harder for women. In November 2015, a regional forum (meeting) was held in Cambodia to focus on improving eye health for women and girls. Knowledge from the forum and from other effective models will be used to produce a guide for good practice. The forum highlighted five practical ways to improve services for women and girls.

1. **Ensure services are community-based.** Screening and appropriate treatment should be provided close to home or in the workplace. Outreach services should be tailored to the specific needs of women and girls and be organised at a time and location suitable to maximise their participation. Schools and market places are two possibilities, but the best ideas and advice will come from women themselves – therefore encourage female community representatives to work with you in programme design.
2. **Tap into the expertise of others.** Partnerships with women's organisations, the women's agency or ministry in the government, maternal and child health services, gender-focused NGOs and microfinance networks can strengthen and more effectively target programmes for women and girls. In Cambodia, the Fred Hollows Foundation is working with the Ministry of Women's Affairs to deliver a project to remove the barriers faced by women.
3. **Disaggregate and analyse data.** Disaggregating (or splitting) clinical data by gender and age is critical – just collecting the figures is not sufficient. Some organisations already disaggregate data by gender for analysis, but it is important that all eye care organisations should do so. Data should be carefully analysed and compared to the demographics of the community. Are there gender differences in the number of patients? Why do they exist? Do the numbers vary on certain days or in different locations? How does the gender balance of staff members affect the balance of patients? Increasingly, donors expect this data and expect to see gender issues addressed.^4^
4. **Create opportunities for women across the eye health workforce, and support them.** Although this varies according to cultural contexts and regions, the gender of eye health workers can affect access to services by female patients. Female eye health professionals need to be supported and mentored. Women often have to work extra hard to negotiate for resources to do their clinical work and must balance family pressures. They also face a greater risk of overt and subtle discrimination and violence in the workplace. Managers of programmes, services, clinics and hospitals need to be attentive and responsive to ensure female workers are employed, retained and promoted.
5. **Reach higher.** Our approach should be based on equity, not just equality. In many contexts, it is not enough for service data to report an equal 50/50 split between men and women. Blindness prevalence is generally higher for women because they have a longer life expectancy and are more likely to experience non-communicable diseases such as cataract and communicable diseases such as trachoma. Some organisations set firm targets that encourage services to reach a higher number of women than men.
